# Vapor-liquid equilibrium of water-hydrogen mixtures: A review of experimental data and modeling with a Cubic-Plus-Association Equation-of-State

**DOI:** 10.1371/journal.pone.0332157

**Published:** 2025-12-29

**Authors:** Joachim Moortgat

**Affiliations:** School of Earth Sciences, The Ohio State University, Columbus, Ohio, United States of America; China University of Petroleum Beijing, CHINA

## Abstract

Interest in subsurface hydrogen storage and geological hydrogen exploration has grown in recent years. These processes generally involve two-phase, multi-component species transport, e.g. for hydrogen and water, and require accurate phase behavior models under varying temperatures and pressures. We compile experimental data spanning 0–200^∘^C and up to 400 bar, revealing non-ideal $H_{2}$–$H_{2}O$ behavior, such as a non-monotonic solubility trend with temperature. To model vapor-liquid equilibrium (VLE) compositions, we use the Cubic-Plus-Association (CPA) equation-of-state (EoS), which effectively captures hydrogen bonding effects. A single temperature-dependent binary interaction coefficient allows accurate reproduction of experimental data across all phases. In contrast, the cubic Peng-Robinson (PR) EoS lacks key molecular interactions and performs poorly, especially for the vapor phase. We also provide a polynomial parameterization of VLE compositions for easy use by hydrogen energy stakeholders. Our results offer a robust framework for hydrogen storage modeling and practical applications.

## Introduction

The global imperative to transition toward carbon-free energy resources has intensified research and industrial efforts around alternative fuels. Among these, hydrogen ($H_{2}$) stands out as a versatile energy carrier with the potential to help decarbonize multiple sectors, including transportation, power generation, and industrial processes [1,2]. Currently, worldwide hydrogen demand exceeds 100 million metric tons per year [3], supporting an industry estimated by different industry research groups and academia to be in the range of $125-$250 billion in 2024 [3–5] and expected to grow rapidly in the next decades. Hydrogen finds extensive use in oil refining, ammonia synthesis, metallurgy, and other chemical processes, and is being explored to help decarbonize transportation, mining, agriculture, airports, and energy generation [4].

Unfortunately, the vast majority of hydrogen (~95–98%) is currently produced from fossil fuels, primarily via steam methane reforming (SMR) [6]. Such processes are energy-intensive, emit large quantities of ${CO}_{2}$, and often rely on non-renewable feedstocks, leading to hydrogen commonly termed “gray” when it originates from methane without carbon capture [7]. “Blue” hydrogen refers to production schemes that employ carbon capture and storage (CCS) to reduce ${CO}_{2}$ emissions. “Green” or “gold” hydrogen refers to sources or generation processes that do not have any associated ${CO}_{2}$ emissions, the most common of which today is electrolysis using renewable electricity. Converting such electricity to hydrogen allows for energy storage given the intermittent nature of, e.g., wind and solar power. Note, though, that electrolysis transforms one form of carbon-free energy into another at less than 100% efficiency and at high production costs.

Recently, there has been heightened attention toward an entirely different and potentially revolutionary source of gold hydrogen: naturally occurring geologic hydrogen reservoirs [8,9]. The first notable example was accidentally discovered in Bourakébougou, Mali, where a village well tapped into a hydrogen-rich gas reservoir [10]. Subsequent investigations suggested that hydrogen in this region is produced through serpentinization and other rock–fluid interactions [11,12], revealing the potential for untapped geological hydrogen resources.

While no geologic hydrogen has yet been produced commercially at scale, we point out that the current total volume of annual global hydrogen production is equivalent to a single medium-sized natural gas reservoir [13]. Put differently, the discovery and succesfull production of even a single geologic hydrogen accumulation in a formation pore volume similar to a medium-sized natural gas reservoir has the potential to double the global supply of hydrogen, and all such hydrogen would be “gold.”

In addition to antropogenic hydrogen generation and prospecting for geologic hydrogen accumulations, another industry is emerging related to the subsurface *storage* of hydrogen, analogous to natural gas storage practices. Hydrogen could be stored in, e.g, depleted hydrocarbon reservoirs, saline aquifers, or salt caverns to help balance seasonal demand fluctuations and bolster energy security [14–16].

Both the naturally occuring geochemical production of hydrogen (through, e.g., serpentinization) and the storage of hydrogen in aquifers involve single- or two-phase systems of, primarily, $H_{2}O$ and $H_{2}$ in porous media. To understand geochemical water-rock reactions (including mineral precipitation or dissolution), the importance of microbial processes, the first occurence of gas bubbles as a result of serpentinization reactions, the ‘loss’ of stored $H_{2}$ due to dissolution into an aqueous phase, the fraction of $H_{2}O$ to expect in produced hydrogen-rich gas, molecular diffusion [17,18], and other critical associated processes, a first prerequisite is to robustly model the thermodynamic phase behavior of $H_{2}O$-$H_{2}$ mixtures.

Specifically, predicting the composition of hydrogen dissolved in water and the fraction of water in the hydrogen-rich gas phase requires reliable vapor–liquid-equilibrium (VLE) data and robust thermodynamic models. These phase compositions vary non-linearly with temperature and pressure, which in subsurface environments translate to depth under hydrostatic or lithostatic conditions. The temperature dependence, in particular, exhibits a counter-intuitive non-monotonic behavior, which cannot be predicted without a rigorous equation-of-state (EoS). Surprisingly, despite the growing relevance of hydrogen and the complexity of water–hydrogen interactions, the amount of published experimental data and validated physical models for $H_{2}O$-$H_{2}$ mixtures is remarkably limited.

In this work, we contribute to narrowing these knowledge gaps by compiling available experimental data on water–hydrogen VLE from historical and more recent sources. We then present a rigorous thermodynamic modeling approach based on the Cubic-Plus-Association (CPA) equation-of-state [19–22]. As the name suggests, CPA contains a cubic part, the Peng-Robinson EoS [23], which describes the physical interactions between molecules. The Peng-Robinson EoS is highly accurate and widely used for pure hydrocarbon mixtures. However, a purely cubic EoS like Peng-Robinson is insufficient to describe mixtures that include self-associating polar molecules, such as $H_{2}O$, and also does not capture the behavior of pseudo-associating molecules such as $CO_{2}$, $H_{2}S$, and $CH_{4}$. The CPA EoS was developed to accurately model such behavior through additional interaction terms [19] and used in reservoir simulations [21] to model complex multi-phase multicomponent problems related to $CO_{2}$ storage [24–26], enhanced oil recovery [22], and natural gas leakage into groundwater [27].

The CPA EoS requires only a single parameter that needs to be adjusted: the binary interaction coefficient (BIC) between $H_{2}$ and $H_{2}O$. We develop a minimization algorithm to obtain the optimal BIC that best fits all compiled experimental data from the literature for $H_{2}O$-$H_{2}$ mixtures, which span a wide range of temperatures (0 to $\sim 200^{\circ }$C) and pressures (1 to ~ 400 bar). Once the BIC is constrained by experimental data, CPA provides an accurate thermodynamic model for all temperature and pressure conditions, and thus depths, that may be encountered in engineering either hydrogen storage or the production of geological hydrogen.

In some works in the literature, and even some widely used commercial reservoir simulators, cubic EoS have been used even for mixtures that contain water [28–30]. Often, different BIC values are used for the aqueous and non-aqueous phases to match experimental data. In fact, there is a large literature on numerous modifications of the PR-EoS to compensate for the limitations of the purely cubic relationship (reviewed in [29]). However, the cubic EoS do not provide a theoretical basis for the interactions involving polar molecules. This approach is merely curve fitting and may result in BIC values <–1 that turn van der Waals attractions into a repulsive force instead. We demonstrate that this is indeed the case for $H_{2}$ – $H_{2}O$ mixtures at low temperatures and that even when separate BICs are optimized for each phase, the PR EoS performs poorly. A robust EoS is essential to model the full thermodynamics, such as phase-stability analyses, phase-split computations, and derived properties like densities, compressibilities of the mixture, partial molar volumes, and so forth. However, the CPA EoS is highly non-linear and rather involved to implement for efficient computations. For stakeholders who are primarily interested in just the VLE equilibrium compositions, i.e. $H_{2}$ solubility in water and $H_{2}O$ fractions in the gas phase, we also derive a more practical polynomial parametrization that can provide especially the former with comparable accuracy.

We use our VLE models to illustrate the non-monotonic dependence of hydrogen solubilities on temperature and its implications for the non-trivial $H_{2}$ – $H_{2}O$ behavior as a function of depth under hydrostatic equilibrium and for different geothermal gradients.

The results presented in this work aim to support the rapidly growing hydrogen sector, from exploration and characterization of natural hydrogen reservoirs to the design and optimization of underground hydrogen storage strategies.

## Compilation of literature VLE experimental data

Hydrogen gas is challenging in laboratory experiments due to its small molecular size (easy to leak), and combustive and corrosive nature. Perhaps because of that, remarkably little phase behavior data for hydrogen are available in the literature. The few exceptions are from nearly a century ago and their accuracy is sometimes hard to judge, involving, e.g., corrections to account for nitrogen impurities, and with results expressed in long deprecated unit systems. A recent overview of experimental data and their temperature and pressure ranges can be found in [31,32], including [33,34]. Available literature data and their accuracy were reviewed in 1981 by the International Union of Pure and Applied Chemistry (IUAC), with historic data converted to standard unit systems [35]. Another compilation of these historic experimental data can be found in the Supplemental Information of [36]. In this work, we use the curated data, expressed as molar fractions, summarized for convenience in Table S1 in the Supplemental Information.

We denote the molar fraction of hydrogen in the aqueous phase by ${H_{2}}_{w}$ and the molar fraction of water in the vapor phase by ${H_{2}O}_{g}$. For a two-component system, ${H_{2}O}_{w}=1\;-\;{H_{2}}_{w}$ and ${H_{2}}_{g}=1\;-\;{H_{2}O}_{g}$, so ${H_{2}}_{w}$ and ${H_{2}O}_{g}$ fully specify both phase compositions. In some cases, a measured ${H_{2}}_{w}$ solubility from one paper and a ${H_{2}O}_{g}$ from another paper are for either temperatures or pressures that are not identical but very close. In Table S1, we have grouped such data for temperatures and pressures that are within 1% from each other and replaced those by their average. Also, while some papers report experimental data up to >1000 bar, for the purposes of this work we only consider those up to 405 bar, which corresponds to pressures down to hydrostatic depths of ~4 km. The experimental temperatures range from to 273.15 < *T* < 473 K (or $0<T<200^{\circ }$C), which easily covers temperatures from typical geothermal gradients down to ~4 km and deeper.

In total, we compiled 49 experimental data points for ${H_{2}O}_{g}$ and measurements of ${H_{2}}_{w}$ at 89 unique temperature-pressure conditions.

The range of experimental data, which will be matched by model predictions, should cover most conditions relevant to hydrogen storage or generation (perhaps short of steam processes).

## Methodology

In this section, we describe the CPA, PR and other EoS as well as our procedures for fitting each relation to the experimental data.

### Thermodynamic equations of state

An equation of state (EoS) describes the thermodynamic phase behavior of a fluid phase (labeled by α) as the temperature ($T_{\alpha }$), pressure ($p_{\alpha }$), and composition (molar fractions $x_{\alpha ,i}$) are varied. In subsurface flow problems, phases α can include water or aqueous (α=w), gas (α=g), multiple liquid phases of, e.g., hydrocarbon oils, nearly solid asphaltenes, light or dense non-aqueous phase liquids (LNAPL, DNAPL), etc. In this work, we only consider aqueous and gas phases consisting of molecular species $H_{2}O$ and $H_{2}$, such that the species label in $x_{\alpha ,i}$ is i=1,2 and by definition $x_{\alpha ,2}=1-x_{\alpha ,1}$.

An EoS can often be written in the canonical form:

$$p_{\alpha }V_{\alpha }=Z_{\alpha }N_{\alpha }RT,\;\text{or}\;p_{\alpha }=Z_{\alpha }c_{\alpha }RT,$$ (1)

in which *R* is the universal gas constant, $V_{\alpha }$ is the volume, $N_{\alpha }$ is the number of moles, and $c_{\alpha }$ is the molar density. For $Z_{\alpha }=1$, one recognizes the familiar ideal gas law. For $Z_{\alpha }\neq 1$, $Z_{\alpha }$ describes all the non-idealities of the fluids. $Z_{\alpha }$ is called the compressibility factor.

For any EoS to fully describe the behavior of a fluid over a wide range of temperature and pressure conditions (when such conditions are of interest), it is important that the EoS includes all the pertinent molecular interactions.

### Peng–Robinson EoS

As an example, the Peng–Robinson (PR) EoS [23], is highly accurate and widely used to describe mixtures of non-polar molecules, e.g. in modeling hydrocarbon oil and gas phases. Peng-Robinson is a cubic EoS:

$$Z_{\alpha }^{3}-(1-B_{\alpha })Z_{\alpha }^{2}+(A_{\alpha }-3B_{\alpha }^{2}-2B_{\alpha })Z_{\alpha }-(A_{\alpha }B_{\alpha }-B_{\alpha }^{2}-B_{\alpha }^{3})=0,$$ (2)

which can also be arranged as

$$Z_{\alpha }=\frac{Z_{\alpha }-B_{\alpha }-A_{\alpha }Z_{\alpha }}{Z_{\alpha }^{2}+2B_{\alpha }Z_{\alpha }-B_{\alpha }^{2}},$$ (3)

which will be useful below, denoting $Z_{pr,\alpha }$ as the right-hand-size of Eq 3.

The dominant molecular interactions considered in the PR EoS are the physical van der Waals interactions, which are represented by $A_{\alpha }$, and the finite volume occupied by molecules, described by $B_{\alpha }$. $A_{\alpha }$ and $B_{\alpha }$ are derived from the *A*_*i*_ and *B*_*i*_ for pure components ($H_{2}O$ and $H_{2}$ in this work) and the binary interaction coefficients (BIC) *k*_*ij*_ using van der Waals mixing rules [37]:

$$A_{ij}=(1-k_{ij})\sqrt{A_{i}A_{j}},$$ (4)

$$A_{\alpha }=\sum_{i=1}^{2}\sum_{j=1}^{2}x_{\alpha ,i}x_{\alpha ,j}A_{ij},$$ (5)

$$B_{\alpha }=\sum_{i=1}^{2}x_{\alpha ,i}B_{i}.$$ (6)

Critically, the binary interaction coefficients *k*_*ij*_ can be temperature dependent and cannot be derived from first principles. They are the primary parameters that need to be fitted to experimental data to complete an EoS model.

### Cubic Plus Association EoS

When a fluid contains polar molecules (like $H_{2}O$, but also asphaltenes and other species), there are additional inter-molecular interactions that cannot be neglected from an accurate thermodynamic model. Water molecules have a permanent dipole moment and self-associate due to hydrogen bonding. Certain other molecules, like $CO_{2}$, $CH_{4}$, and $H_{2}S$, do not have a permanent dipole moment but a temporary polar-induced moment in the presence of water. Such molecules do not self-associate but can *cross-*associate with $H_{2}O$, which again alters the non-ideal behavior of such fluid mixtures. The CPA EoS was developed by Li and Firoozabadi [19], who demonstrated the importance of the self- and cross-association terms in modeling experimental data different fluid mixtures.

The CPA EoS can be written as:

$$Z_{\alpha }=Z_{pr,\alpha }+\left(1+\frac{B_{\alpha }}{B_{\alpha }-8Z_{\alpha }-3B_{\alpha }}\right)\sum_{i=1}^{2}x_{\alpha ,i}\sum_{j=1}^{2}\eta_{ij}(\chi_{\alpha ,ij}-1).$$ (7)

Association occurs between opposite pairs of hydrogen-bond donor sites (*j* = 1) and hydrogen-bond acceptor sites (*j* = 2). The number of donor or acceptor sites (*j*) for each species *i* is denoted by $\eta_{ij}$. As an example, water has two donor and two acceptor sites ($\eta_{w,1}=\eta_{w,2}=2$).

The fraction of sites *j* on species *i* in phase α that are free (i.e., not occupied by hydrogen bonds) are given by $\chi_{\alpha ,ij}$. Their computation can be highly complex for the most general cases but can be simplified for our problem by assuming that the bonding between donor and acceptor sites is symmetric, such that $\chi_{\alpha ,ij}=\chi_{\alpha ,ji}$. Since only water self-associates, we only need components $\chi_{\alpha ,w}=\chi_{\alpha ,w,w}$ and $\chi_{\alpha ,i}=\chi_{\alpha ,w,i}=\chi_{\alpha ,i,w}$ when considering cross-association. These site fractions can be computed from:

$$\chi_{\alpha ,w}=\frac{Z_{\alpha }}{Z_{\alpha }+2\sum_{i=1}^{2}x_{\alpha ,i}\chi_{\alpha ,i}\Delta_{\alpha ,w,i}},$$ (8)

$$\chi_{\alpha ,i}=\frac{Z_{\alpha }}{Z_{\alpha }+2x_{\alpha ,w}\chi_{\alpha ,w}\Delta_{\alpha ,w,i}},$$ (9)

where $\Delta_{\alpha ,w,i}$ represents the self- and cross-association strengths, respectively:

$$\Delta_{\alpha ,w,w}=\frac{8Z_{\alpha }^{2}B_{\alpha }-8Z_{\alpha }(B_{\alpha }-4Z_{\alpha })}{3\kappa p_{\alpha }RT}\exp\left(\frac{\epsilon }{k_{B}T}-1\right),$$ (10)

$$\Delta_{\alpha ,w,i}=s_{i}\Delta_{\alpha ,w,w},\;i\neq w,$$ (11)

where *s*_*i*_ is a cross-association parameter between water and species *i*, and ϵ and κ are energy and volume parameters, respectively, of the self-association of water, given by:

$$\epsilon =1738.4/k_{B}\;\text{K},$$ (12)

$$\kappa =1.8015\times 10^{-3}\;\text{L/mol},$$ (13)

with *k*_*B*_ the Boltzmann constant.

Note that CPA does not model hydrogen bonds as discrete complexes. The contribution to the Helmholtz free energy from association decreases with increasing temperature due to the Boltzmann factor $\exp\left(\frac{\epsilon }{k_{B}T}\right)$. Therefore, at high temperatures, the extent of hydrogen bonding predicted by CPA naturally diminishes, reflecting the thermal destabilization of H-bond networks. No explicit temperature cutoff is imposed; rather, association effects emerge or vanish as a consequence of the thermodynamic formulation.

The above description is the general form that allows for both self-association and cross-association. Because $H_{2}$ is a homonuclear diatomic molecule (two identical atoms sharing electrons), there are no significant partial charges or lone pairs to act as hydrogen-bond donors or acceptors. Hence, in typical molecular modeling approaches, $H_{2}$ is considered to have zero donor and zero acceptor sites for hydrogen bonding. In that case, the cross-association parameter *s*_*i*_ = 0. As a result, $\Delta_{\alpha ,w,i}=0$, $\chi_{\alpha ,i}=1$ and

$$\chi_{\alpha ,w}=\frac{Z_{\alpha }}{Z_{\alpha }+2x_{\alpha ,w}\chi_{\alpha ,w}\Delta_{\alpha ,w,w}},$$ (14)

which can be solved analytically. The positive solution is

$$\chi_{\alpha ,w}=\frac{\sqrt{Z_{\alpha }^{2}+8x_{\alpha ,w}\Delta_{\alpha ,w,w}Z_{\alpha }}-Z_{\alpha }}{4x_{\alpha ,w}\Delta_{\alpha ,w,w}}.$$ (15)

Cross-association *was* considered together with BIC in a two-parameter regression to fit the experimental data, but the optimal cross-association strengths *s*_*i*_ were indeed low (of the order of 10^−9^) and did not exhibit a monotonic dependence on temperature. For conciseness, only results without cross-association will be shown in the Results and Discussion sections.

Despite the simplifications for the two-component $H_{2}O$-$H_{2}$ system, it is clear that Eq 15 together with Eq 7 is still highly non-linear. In fact, it cannot be written as a closed-form polynomial of known order, i.e. even the number of solutions is unknown a priori. Efficient iterative solution techniques were presented in an earlier work [22], which also provides further details on how the CPA EoS is incorporated into phase stability analyses (minimizing the Gibbs free energy) and phase-split computations (guaranteeing the equality of chemical potentials or fugacities for each species in each phase).

In terms of broader applicability of the CPA EoS, its accuracy has previously been validated against experimental data for pressures up to 1000 bar and temperatures of 500^∘^ K for mixtures involving $H_{2}O$/$C_{1}$, $H_{2}O$/$C_{2}$, $H_{2}O$/$CO_{2}$, $H_{2}O$/$H_{2}S$, $H_{2}O$/1-hexene, $H_{2}O$/1-octene, $H_{2}O$/1-decene, $H_{2}O$/benzene, $H_{2}O$/ethylbenzene, $H_{2}O$/m-diethylbenzene, $H_{2}O$/1-methylnaphthalene, $H_{2}O$/1-ethyl- naphthalene, and $H_{2}O$/$C_{1}$/$CO_{2}$/$H_{2}S$ in two and three phases [19]. CPA does *not* account for ionic species, even though we know that solubilities often decrease with brine salinity. Incorporating such effects into an accurate EoS is an active area of ongoing research. The accuracy of CPA for $H_{2}O$-$H_{2}$ mixtures in near-critical or supercritical conditions is also not explored here because we do not have experimental data at $T\geq T_{c}=647^{\circ }$K temperatures.

### Other EoS approaches

Other equations of state that have been used to model hydrogen-water mixtures [32] are the Schwartzentruber and Renon modified Redlich–Kwong (SR-RK) [38–41] and the perturbed-chain statistical associating fluid theory (PC-SAFT) EoS [42–49]. Both EoS were able to match the experimental data considered with RMSE errors of 4% – 5% [32]. A less desirable aspect of SR-RK is that it has many free parameters that need to be adjusted. For PC-SAFT, the following temperature dependencies were considered in the binary interaction coefficients [32]:

$$k_{ij}=a_{ij}+\frac{b_{ij}}{T_{r}}+c_{ij}\ln T_{r}+d_{ij}T_{r}+e_{ij}T_{r}^{2},$$ (16)

in which $a_{ij},b_{ij},c_{ij},d_{ij},e_{ij}$ are fitted to experimental data (finding $d_{ij}=e_{ij}=0$ for $H_{2}O$ – $H_{2}$). A different version of group contribution PC-SAFT (GC-PC-SAFT) EoS has also been used to model mixtures of hydrogen and hydrocarbons [50].

Given the convenience and simplicity of the PR-EoS, it is sometimes also used for problems that involve polar molecules like water. In an attempt to match experimental phase behavior data, different BICs *k*_*ij*_ are used for each phase. While this approach is fast and may provide reasonable results in a narrow temperature-pressure-composition range where the *k*_*ij*_ have been adjusted, this approach has no physical basis and cannot be generalized to arbitrary multicomponent mixtures. Moreover, it is less elegant and more error prone than a thermodynamic model that treats each phase the same, such that phase identification is not necessary.

### Implementation of optimized $H_{2}$ – $H_{2}O$ VLE models in this work

#### CPA EoS.

Our primary, most physically robust, model for $H_{2}$ – $H_{2}O$ vapor-liquid-equilibrium is the CPA EoS presented in section Cubic Plus Association EoS. We assume that there is no polar-induced cross-association between $H_{2}O$ and $H_{2}$ but we do consider the important self-association of water. Because we only have pure components, there is no need to tune critical properties of pseudo-components. We use the same critical properties for $H_{2}$ and $H_{2}O$ as in earlier works [32], summarized for convenience in Table 1. The only parameter in CPA-EoS that is tuned to experimental data is the binary-interaction-coefficient (BIC) between $H_{2}$ and $H_{2}O$. We demonstrate in the Results section that a linear dependence on temperature suffices, i.e. even simpler than for PC-SAFT in Eq 16.

**Table 1:** Molecular Properties of Water (H_2_O) and Hydrogen (H_2_).

Property	Water (H_2_O)	Hydrogen (H_2_)
Acentric factor (ω)	0.3440	-0.216
Critical temperature (*T*_*c*_)	647.29 K	33.0 K
Critical pressure (*P*_*c*_)	220.9 bar	12.9 bar
Molecular weight (*M*_*w*_)	$18.02\;{g\;mol}^{-1}$	$2.0159\;{g\;mol}^{-1}$

Phase stability and phase-split computations are performed with our in-house research reservoir simulator, *Osures*, whose features have been described in numerous earlier publications that have already been cited above. To tune the EoS description by fitting to experimental data, we developed a python wrapper for this work. A set of temperature, pressure, BIC, and cross-association strength (when considered) is fed to *Osures*, which in turn returns the hydrogen fraction in the aqueous phase ${H_{2}}_{w}$ and the ${H_{2}O}_{g}$ fraction in the gaseous phase. We then use convenient open source python packages like scipy.optimize.least_squares to run the minimization/optimization with default parameters.

The cost (error metric) that is minimized is the sum of the squares of residuals:

$$cost=\sum_{i=1}^{n_{w}}{\left({H_{2}}_{w,exp,i}-{H_{2}}_{w,CPA,i}\right)}^{2}+\sum_{i=1}^{n_{g}}{\left({H_{2}O}_{g,exp,i}-{H_{2}O}_{g,CPA,i}\right)}^{2}$$ (17)

which is closely related to the root-mean-squared-errors (RMSE):

$$RMSE({H_{2}}_{w})=\frac{1}{n_{w}}\sum_{i=1}^{n_{w}}{\left({H_{2}}_{w,exp,i}-{H_{2}}_{w,CPA,i}\right)}^{2},$$ (18)

and

$$RMSE({H_{2}O}_{g})=\frac{1}{n_{g}}\sum_{i=1}^{n_{g}}{\left({H_{2}O}_{g,exp,i}-{H_{2}O}_{g,CPA,i}\right)}^{2},$$ (19)

where *n*_*w*_ is the number of experimental data points for ${H_{2}}_{w}$ and *n*_*g*_ for ${H_{2}O}_{g}$ and $n_{w}\neq n_{g}$. We note that for some temperatures only $H_{2_{w,exp}}$ or only ${H_{2}O}_{g,exp}$ are available, in which case we only consider those residuals.

In the minimization scheme, any *overall* multiplicative factor on the errors does not affect the results (e.g. dividing by number of data-points or normalizing the squared residuals). We did experiment with giving each of the cost terms different weights because the magnitudes of $H_{2_{w,exp}}$ are up to a factor of ~40 smaller than those of ${H_{2}O}_{g,exp}$ at certain temperatures. We also tested an approach of normalizing each residual by the experimental value (i.e., $\left((H_{2_{w,exp}}-H_{2_{w,CPA}})/H_{2_{w,exp}}\right){\;}^{2})$. However, after fitting a linear relationship, $k_{ij}=a_{ij}\times T$, to the optimized BICs, the difference in *a*_*ij*_ for all approaches was negligible.

Besides the RMSE, we also consider the Average Absolute Deviations (AAD):

$$AAD({H_{2}}_{w})=\frac{1}{n_{w}}\sum_{i=1}^{n_{w}}\left|{H_{2}}_{w,exp,i}-{H_{2}}_{w,CPA,i}\right|,$$ (20)

and similarly for AAD (${H_{2}O}_{g})$. We also present the coefficients of correlation (R^2^ scores):

$$R^{2}({H_{2}}_{w})=1-\frac{\sum_{i=1}^{n_{w}}{\left({H_{2}}_{w,exp,i}-{H_{2}}_{w,fit,i}\right)}^{2}}{\sum_{i=1}^{n_{w}}{\left({H_{2}}_{w,exp,i}-\overset{―}{{H_{2}}_{w,exp}}\right)}^{2}},$$ (21)

in terms of the mean experimental concentrations

$$\overset{―}{{H_{2}}_{w,exp}}=\frac{1}{n_{w}}\sum_{i=1}^{n_{w}}{H_{2}}_{w,exp,i},$$ (22)

and again similarly for *R*^2^ (${H_{2}O}_{g})$

Note that $0<{H_{2}}_{w}<1$ and $0<{H_{2}O}_{g}<1$ are intrinsically dimensionless and normalized molar fractions, and therefore the RMSE and AAD are also already normalized errors. Because the molar fractions can be quite small, we multiply molar fractions by a factor 1000 and report all results in the following section as per mil () for clarity. We acknowledge a potential source of confusion: when a RMSE error is reported as a certain (or similar when using %), this is relative to the full range of possible concentrations (i.e. up to 100%). We could instead normalize errors by either the mean or the range of experimental values (both common approaches), but this range depends on the range of pressures measured at each temperature (which is not the same), it would be different if we plot compositions versus temperature instead of pressure (as we do in one example), etc. A less ambiguous approach is to normalize by the full range of *all* measured conditions. For ${H_{2}O}_{g}$ this range is up to ~25%, whereas for ${H_{2}}_{w}$ this is up to ~0.5%. One may therefore choose to multiply our reported RMSE and AAD errors for ${H_{2}O}_{g}$ by a factor ~4 and those for ${H_{2}}_{w}$ by a larger factor of up to ~200 to obtain errors relative to the measured range.

#### PR EoS.

Because the Peng-Robinson EoS [23], as well as other cubic EoS, is so widely used and sometimes even for water-containing mixtures, we will also present optimized results for this EoS. When a single optimized BIC is used for all phases, errors are exceedingly high. We therefore use the common approach, when applying PR EoS to water-containing mixtures, of using different temperature dependent BIC for each phase. Using the same procedure as described above, we first minimize the residuals for just ${cost}_{w}=\sum_{i=1}^{n_{w}}\left({H_{2}}_{w,exp,i}-{H_{2}}_{w,CPA,i}\right){\;}^{2}$ to find an optimal BIC_*w*_ and next we minimize ${cost}_{g}=\sum_{i=1}^{n_{g}}\left({H_{2}O}_{g,exp,i}-{H_{2}O}_{g,CPA,i}\right){\;}^{2}$ to find the optimal BIC_*g*_.

#### Parametric VLE predictions.

For researchers and other stakeholders who may not have access to EoS-based phase-stability and phase-split softwares, yet need to estimate hydrogen solubilities at different subsurface depths or water fractions in produced hydrogen gas streams, we also perform various regressions to find a parametric representation that can fit the experimental data over a wide range of temperatures and pressures. As we demonstrate below, a low-order polynomial approximation can match the measured ${H_{2}}_{w}$ with acceptable accuracy. This can also be useful to provide initial guesses in iterative solution schemes for a full EoS thermodynamic model. However, attempts to match ${H_{2}O}_{g}$ with numerous combinations of polynomials, exponentials, logarithms, reciprocals, and square roots, did not achieve comparable accuracies. We will present the most generalizable polynomial approximation, despite its relatively low accuracy.

## Results

In the following, we present model predictions, and comparisons to experimental data, for the CPA EoS, the PR EoS, and a parametric representation.

### CPA-EoS predictions

**Fig 1:**
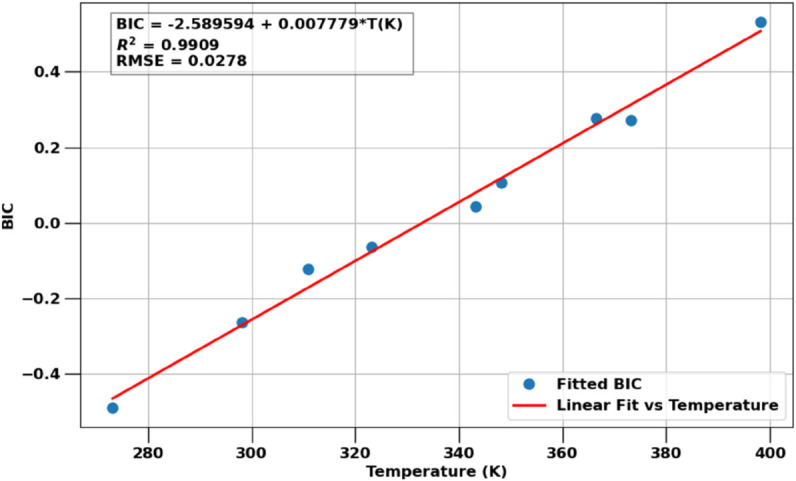
Optimized BICs, *k*_*ij*_, for the CPA EoS that best match experimental data (symbols). The BIC are temperature dependent and show a clear linear trend. A fitted linear model is shown as a solid line together with the coefficient of correlation and RMSE of the fit. Note that the same BIC is used for all phases.

Fig 1 shows the key parameter that is optimized in our regressions to match the CPA EoS to experimental data over a wide range of temperatures and pressures. Specifically, we find the optimal binary interaction coefficients (BIC) for temperatures ranging from 273.15<*T*<473 K ($0<T<200^{\circ }$C). The BICs show a clear linear trend with temperature, fitted as

$$k_{ij,CPA}=-2.6+7.8\times 10^{-3}\;T(K).$$ (23)

**Fig 2:**
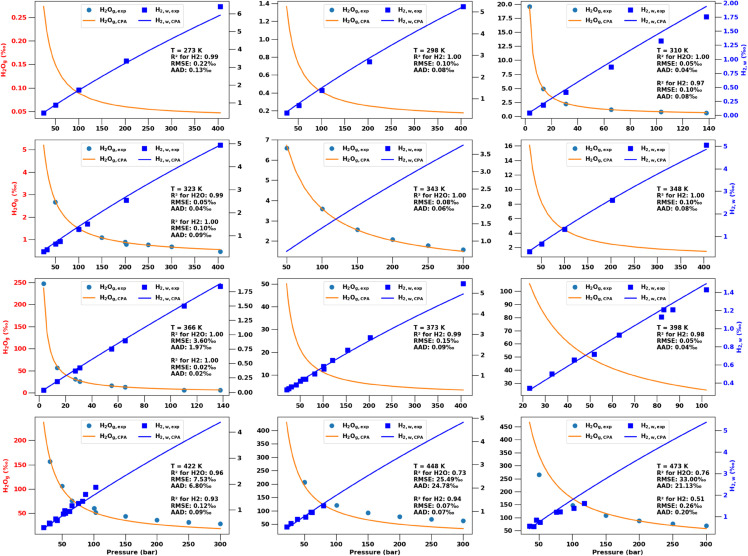
Experimental measurements of hydrogen solubility in water (${H_{2}}_{w,exp}$) and water fraction in the gas phase (${H_{2}O}_{g,exp}$), shown by symbols. Corresponding predictions from the optimized **CPA EoS** are shown by solid lines for ${H_{2}}_{w,CPA}$ and ${H_{2}O}_{g,CPA}$ (using the BICs from Fig 1). All molar fractions (and associated errors) are converted to per mil (). Each panel is for a different temperature and also shows the coefficient of determination, RMSE, and AAD for the CPA model predictions versus experimental data.

Using the temperature dependent BICs from Eq 23, we perform VLE computations for all experimental conditions (plus additional intermediate pressure values) and provide the predictions as well as experimental data points in Fig 2. The figure panels for 12 different temperatures also show various accuracy metrics of how well the CPA EoS model matches the experimental data.

Note that Fig 2 (and similar figures in the sections below) shows the same VLE data as in a conventional *p*-*x*-*y* phase diagram, but in a more clear visualization. Because ${H_{2}}_{w}\approx 0$ and ${H_{2}}_{g}\approx 1$ for the *T*-*p* ranges considered, a standard *p*-*x*-*y* phase diagram would show near vertical lines at those compositional extremes (e.g., as Fig 5 in [32]). By using different vertical axes/ranges for ${H_{2}}_{w}$ and ${H_{2}O}_{g}$, the pressure dependencies are more clear.

### Peng-Robinson EoS predictions

**Fig 3:**
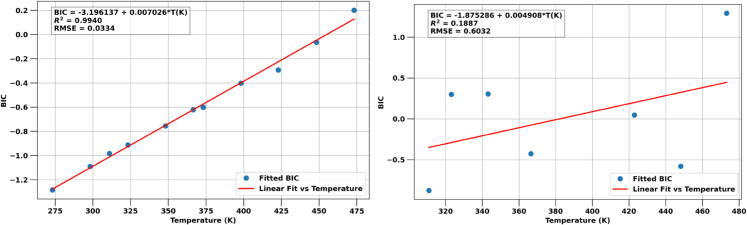
For the PR EoS, different BICs have to be used for each phase. Optimized BICs that best match experimental data (symbols) are shown for the aqueous phase **(left)** and gas phase **(right)**. Fitted linear models are shown as a solid line together with the coefficients of correlation and RMSEs of the fit.

When using the PR EoS, i.e., without accounting for the interactions due polar water molecules, approximating experimental results with the same BIC for all fluid phases is exceedingly inaccurate. We therefore optimize the BICs separately for the aqueous and gas phases, using the experimental data for each phase, independent from each other. Fig 3 summarizes the results from these regressions.

The optimized BICs for the aqueous phase are again clearly linear (the fitted model equations and error metrics are shown in the figure). For the gas phase, the optimal BICs do not exhibit any clear functional dependence on temperature, nor even a monotonic trend. The shown linear fit may be the best generalizable trend (beyond perhaps a constant average BIC).

**Fig 4:**
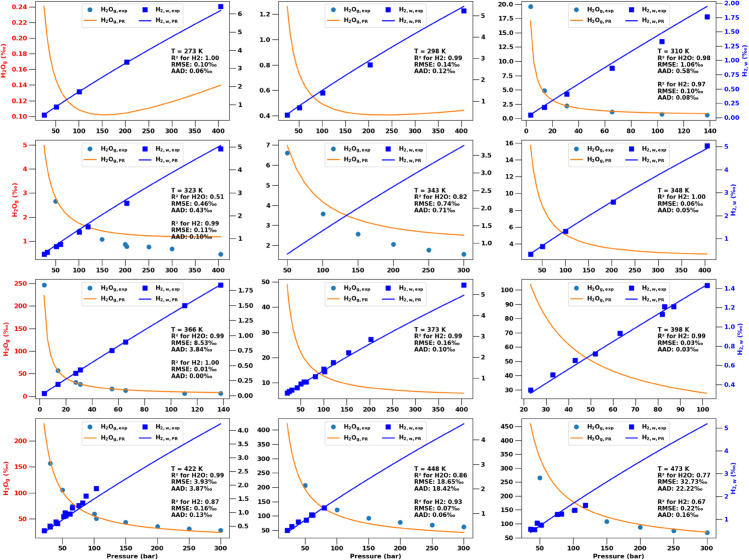
Experimental measurements of ${H_{2}}_{w,exp}$ and ${H_{2}O}_{g,exp}$, shown by symbols. Predictions from the optimized **PR EoS** are shown by solid lines for ${H_{2}}_{w,PR}$ and ${H_{2}O}_{g,PR}$, using different BICs for each phase as illustrated in Fig 3.

In Fig 4 we compare the optimized predictions from the PR EoS to experimental data. We again observe reasonable agreement between model predictions and experimental data for ${H_{2}}_{w}$ but much worse performance for the predictions of ${H_{2}O}_{g}$, as we will discuss further in the Discussion.

### Parameterized model predictions

Finally, we attempt to fit all experimental data with a convenient polynomial, or otherwise parameterized, relation (that is however entirely devoid of a physical underpinning). The objective is to provide anyone interested in subsurface hydrogen-water mixtures with reasonable estimates of hydrogen solubilities in water as well as water fractions in hydrogen-rich gas.

**Fig 5:**
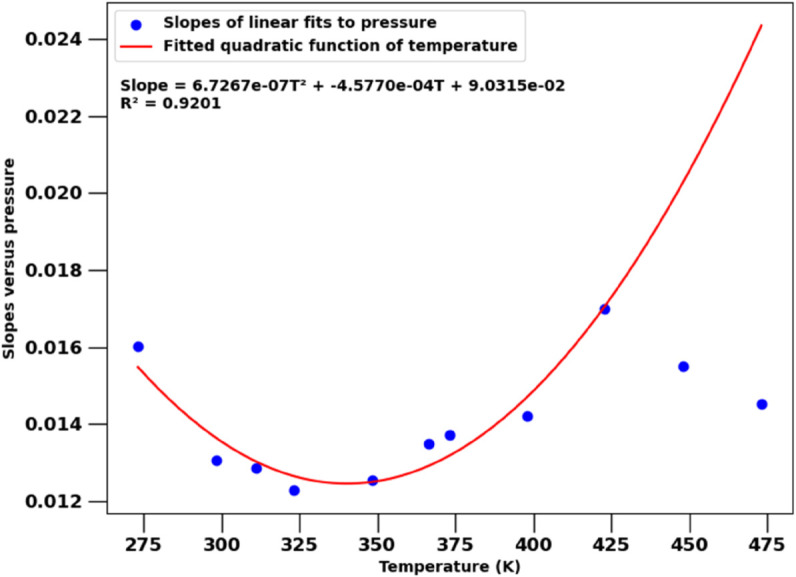
Polynomial approximation finds ${H_{2}}_{w}\approx \beta \left(T\right)\;\times \;p$, with slopes β(T) shown as symbols. Slopes β(T) are fitted as a quadratic function of temperature, shown as a solid line together with its full equation and R^2^ score (excluding the two highest pressure points).

As in the previous section for the PR EoS, we can accurately match the ${H_{2}}_{w}$ because its dependence on temperature and pressure is nearly polynomial. Specifically, the dependence on pressure can be approximated reasonably well by a direct proportionality (${H_{2}}_{w,exp}\approx \beta \times p$), while the slopes β(T) of that relationship exhibit a nearly quadratic dependence on temperature, as illustrated in Fig 5. Because the experimental data at the highest two temperatures appear to be outliers in all analyses, we excluded those slopes from the regression but do show them in all figures.

The fully parameterized relation is:

$${H_{2}}_{w,fit}(T,p)=\left(6.7\times 10^{-7}\;T^{2}-4.6\times 10^{-4}\;T+9.0\times 10^{-2}\right)\times p.$$ (24)

**Fig 6:**
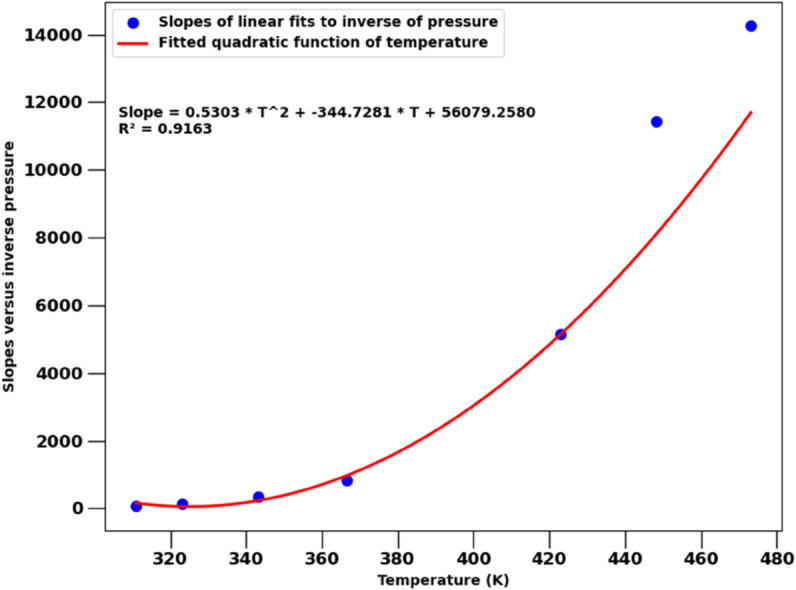
Temperature dependent slopes α(T) of parameterized ${H_{2}O}_{g}\approx \alpha \left(T\right)/p$ (symbols), fitted with a quadratic polynomial α(T) = 0.53 $\times T^{2}$ – 344.73 × T + 56 × 10^3^ (solid line).

The experimental data for ${H_{2}O}_{g}$ cannot be as easily parameterized with similar accuracy. The dependence on pressure follows a clear reciprocal relation (${H_{2}O}_{g}\propto 1/p$). The slopes of those fits increase steeply with temperature, but neither polynomial dependencies (up to 12-th order), nor exponential, or other functional relationships can fit the data well. Overly complex models (like high-order polynomials) are overfitted and generalize poorly even for temperatures within the range of experimental conditions. After much experimentation, a quadratic dependence on temperature (as shown in Fig 6), together with the 1/*p* scaling:

$${H_{2}O}_{g,fit}(T,p)=\left(0.5\;T^{2}-344.7\;T+56\times 10^{3}\right)/p$$ (25)

may be as good as pure curve fitting to experimental data can perform.

**Fig 7:**
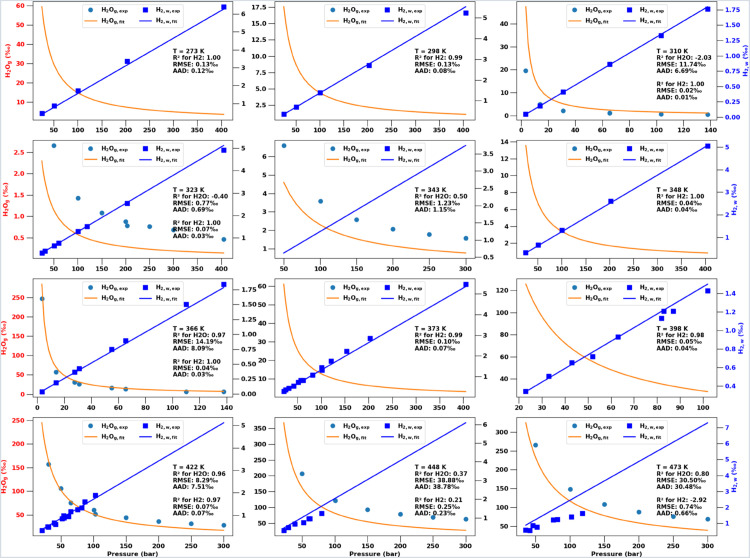
Experimental measurements of ${H_{2}}_{w,exp}$ and ${H_{2}O}_{g,exp}$, shown by symbols. Predictions from an optimized **parameterization** are shown by solid lines, together with coefficients of determination, RMSE, and AAD for the parameterized predictions versus experimental data.

Fig 7 summarizes the predictions of this parameterized model across all experimental data for ${H_{2}}_{w,exp}$ and ${H_{2}O}_{g,exp}$, and the associated error metrics.

## Discussion

Clearly, the CPA EoS is the most physically robust description of the $H_{2}$-$H_{2}O$ VLE phase behavior as it describes all key physical interactions between the molecules. Fig 2 shows that the EoS can predict the compositions of both phases with a high degree of accuracy over a wide range of temperatures and pressures. Only at the highest temperatures do the coefficients of determination drop below *R*^2^ = 0.95. The scatter pattern in the experimental measurements, collated from different literature sources, suggests that some of this may be due to experimental errors. Consider, for example, the non-monotonic trend in data points at 398^∘^K and between 80 and 90 bar in Fig 2. In fact, when ${H_{2}}_{w}\propto p$ is directly proportional (Eq 24), the RMSE, R^2^, and AAD are essentially direct measures of such experimental errors.

To reproduce the $H_{2}$-$H_{2}O$ VLE phase compositions with a cubic EoS like Peng Robinson [23] requires different binary interaction coefficients (BIC) in each phase. Fig 4 suggest that this approach can predict ${H_{2}}_{w}$ with reasonable accuracy. However, note from Fig 3 that this requires BICs for the aqueous phase with BIC_*w*_<−1. Binary interaction coefficients are a measure of the attractive strength of van der Waals forces between molecules. Smaller BICs reflect a weaker attraction, but as Eq 4 shows, BICs <–1 actually turn the van der Waals interaction into a repulsive force, which has no physical basis.

Even with separate optimized temperature dependent BICs for the gas phase, the PR EoS predictions for ${H_{2}O}_{g}$ (Fig 3) are significantly worse than from the CPA EoS (Fig 2). This is also in line with an earlier study that compared predictions from optimized PR and SRK EoS compared to molecular simulations and experimental data [36].

We note that even more corrections have been proposed, and some explored for $H_{2}O$-$H_{2}$ [32], such as extending temperature-dependent alpha-functions with polar parameters [40] or modifying the mixing rules such as those of Wong–Sandler [51] and Huron–Vidal [52], which combine cubic EoS with excess Gibbs energy models (e.g., NRTL [53] or UNIQUAC [54]). These methods all introduce multiple additional empirical parameters, which may be tuned to specific mixtures but lack a consistent physical basis for strongly associating systems like $H_{2}O$-$H_{2}$. In contrast, the CPA EoS provides a thermodynamically rigorous framework that requires only a single binary interaction coefficient and explicitly models hydrogen bonding effects. For clarity and parsimony, we have therefore focused only on comparing CPA to the baseline PR model under typical cubic-EoS usage.

A purely polynomial approximation, devoid of all physics, can provide reasonable predictions for the aqueous compositions (e.g. ${H_{2}}_{w}$), though performs poorly for the vapor compositions. Such a polynomial relation with reasonable accuracy is actually also useful for EoS modeling: equations of state like CPA are highly non-linear and phase stability and phase-split computations involve iterative schemes like successive substitution and Newton-Raphson. Such schemes require initial guesses and converge faster when the initial guesses are better. In other words, a relation like Eq 24 can help speed up large-scale simulations of subsurface $H_{2}$-$H_{2}O$ flow processes, using a rigorous EoS.

**Fig 8:**
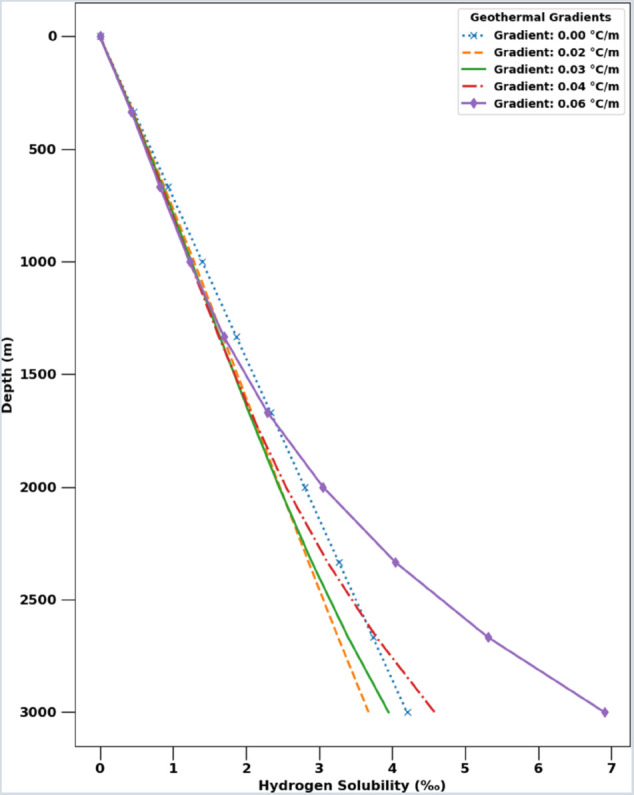
Hydrogen solubility in water versus depth.

One practical example where a simple parameterization like in Eq 24 may be useful is to estimate hydrogen solubility as a function of subsurface depths based only on reasonable assumptions of a hydrostatic pressure gradient and different geothermal temperature gradients. In Fig 8 we provide examples of this for three typical geothermal gradients of 0.02, 0.03 and $0.04^{\circ }$C/m. For illustrative purposes, we also include curves for a high geothermal gradient of $0.06^{\circ }$C/m, as well as for a constant temperature, such that the depth dependence is purely from the hydrostatic pressure and thus varies linearly with depth (Eq 24).

**Fig 9:**
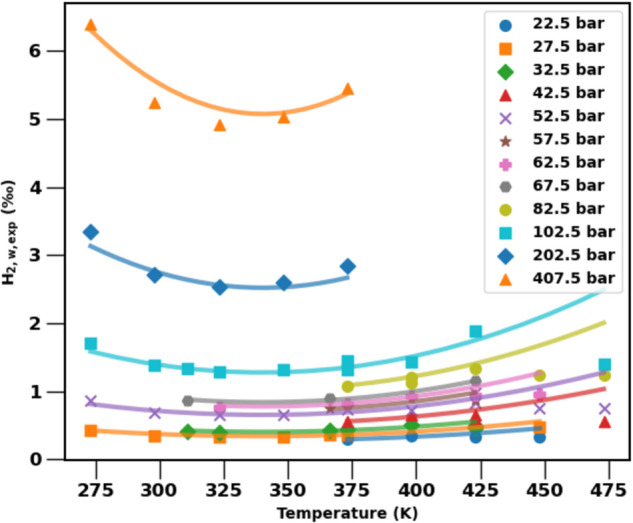
Hydrogen solubility in water plotted versus temperature for different pressures. Pressures are grouped into bins of 5 bar with the average shown in the legend.

The behavior is more interesting for the varying temperature gradients, which result in a non-monotonic dependence of solubilities with depth. This is because the dependence of $H_{2}$ solubility itself depends non-monotonically on temperature. To illustrate this more clearly, in Fig 9 we plot the same experimental data for ${H_{2}}_{w,exp}$ as before but now as a function of temperature instead of pressure. To be more precise, we group the experimental data into 5-bar bins of increasing pressure (with the mid-point pressures shown in the legend) and for each pressure bin, we plot the data versus temperature. Only pressure bins with more than three temperature conditions are shown. The solid lines are the parameterized predictions at the mid-point pressures (and would be similar from the CPA EoS).

Fig 9 demonstrates that at low temperatures hydrogen solubilities exhibit the intuitive behavior of decreasing with temperature. However, beyond a certain temperature, the solubility *increases* with temperature, which reflects the non-ideal behavior of the $H_{2}$ – $H_{2}O$ mixture at subsurface temperatures and pressures. In fact, from Eq 24 we can directly solve for that temperature of minimum hydrogen solubility, which is $T_{min}\approx 340$ K for all pressures. Because this minimum solubility temperature occurs at different depths for different geothermal gradients, the curves in Fig 8 intersect.

### Mixtures with $CO_{2}$ and $CH_{4}$

Subsurface formations targeted for hydrogen storage, or for the production of naturally occurring hydrogen, may not be saturated with 100% $H_{2}O$ but also contain other common molecular species like $CO_{2}$, $CH_{4}$, $H_{2}S$, and sometimes hydrocarbons. In addition, injected hydrogen for storage may also contain small amounts of such species. Here we analyze these additional complexities for the VLE phase behavior of three-component mixtures of $H_{2}O$-$CH_{4}$-$H_{2}$ and $H_{2}O$-$CO_{2}$-$H_{2}$. We are not aware of experimental data of such ternary mixtures, but the accuracy of CPA with cross-association has been demonstrated for binary mixtures of $H_{2}O$-$CH_{4}$ and $H_{2}O$-$CO_{2}$ [19]. Fitted binary interaction coefficients and cross-association strengths for these mixtures were provided in earlier work [22].

As an illustrative example, we consider mixtures of 50 mol% $H_{2}O$ with 50 mol% gas at 323^∘^K and for pressures varying from 10 to 400 bar. The feed gas composition ($H_{2}/(1-H_{2}O)$) is varied as 2%, 20%, 40%, 80%, and 98% of $H_{2}$ with either $CH_{4}$ or $CO_{2}$ making up the remainder (before achieving VLE).

**Fig 10:**
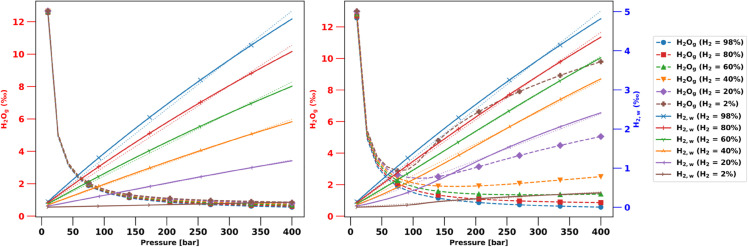
VLE ${H_{2}}_{w}$ and ${H_{2}O}_{g}$ compositions for ternary mixtures with $CH_{4}$ ( left) and $CO_{2}$ ( right) at 323^∘^K. Dashed lines are for linear fits to ${H_{2}}_{w}$ as a function of pressure.

Fig 10 shows the corresponding ${H_{2}}_{w}$ and ${H_{2}O}_{g}$ compositions as before. For the $H_{2}O$-$CH_{4}$-$H_{2}$ mixtures, we see that the vapor composition of $H_{2}O$ is relatively insensitive to the $CH_{4}$-$H_{2}$ fractions in the gas phase. The fraction of dissolved hydrogen, ${H_{2}}_{w}$, reduces linearly with increasing $CH_{4}$ due to competitive dissolution. Quantitatively, denoting ${H_{2}}_{,gf}$ as the hydrogen molar fraction in the gas phase feed before mixing with water, we find that ${H_{2}}_{w}=0.013\times {H_{2}}_{,gf}\times p$. The R^2^ coefficients of the fits are all >99%, but decreasing with increasing $CH_{4}$ fractions. $CH_{4}$ is a weakly cross-association species, which introduces some non-linearity in the pressure dependence, as is evident from the dashed linear fits for increasing $CH_{4}$ compositions.

Cross-association between $CO_{2}$ and $H_{2}O$ is profound and cannot be neglected. The vapor fraction of water, ${H_{2}O}_{g}$, exhibits a particularly non-linear and non-monotonic dependence on pressure for high $CO_{2}$ concentrations (right panel in Fig 10). This behavior is real and has been validated against experimental data [19]. The non-monotonic trend is entirely due to cross-association and cannot be captured by equations of state that do not include the cross-association interactions. The dissolution of $CO_{2}$ in water also varies non-linearly with pressure, which in turn affects the competitive dissolution of hydrogen. At low $CO_{2}$ concentrations, ${H_{2}}_{w}$ still varies linearly with pressure but at higher $CO_{2}$ concentrations the pressure dependence becomes more non-linear. The slopes of the ${H_{2}}_{w}(p)$ pressure dependence also do not scale linearly with ${H_{2}}_{,gf}$ (such a fit has a R^2^ of 78%).

## Conclusions

- In this work we studied the phase behavior of hydrogen-water mixtures at a wide range of temperatures and pressures relevant to subsurface geological formations. Specifically, we compiled experimental measurements of aqueous $H_{2}$ – $H_{2}O$ compositions at 89 unique temperature-pressure conditions and 49 such data points for gas phase compositions for $H_{2}$ – $H_{2}O$ thermodynamic vapor-liquid-equilibrium (VLE). The pressures range from near-atmospheric (3.4 bar) to 405 bar, covering hydrostatic pressures down to ~4 km, and the range of temperatures cover 273.15<*T*<473 K (or $0<T<200^{\circ }$C).
- As a robust thermodynamic model to predict the VLE behavior of hydrogen in the Earth’s subsurface, we tune the cubic-plus-association (CPA) EoS to match these experimental data. Only a single free parameter, the binary interaction coefficient (BIC) between $H_{2}$ and $H_{2}O$ (Eq 4) needs to be adjusted to fully specify this model. Moreover, we find that this BIC is directly proportional to temperature and thus only requires a single proportionality constant. The performance of the CPA EoS in terms of accuracy is likely similar to PC SAFT but requiring even less parameter adjustments [32], and far fewer adjustable parameters than the Schwartzentruber and Renon modified Redlich–Kwong (SR-RK) EoS.
- The widely used Peng-Robinson EoS can be adjusted to model mixtures that contain polar molecules like water by using different BICs for each phase. By following this procedure, we find that the hydrogen solubilities in water can indeed be matched with reasonable accuracy, because its dependence on temperature and pressure is approximately polynomial. However, this is essentially just curve fitting without considering the actual physical interactions. Specifically, such a fit requires BIC values below –1 which imply a repulsive force instead of van der Waals attraction interactions. Moreover, because the vapor compositions do not have a straightforward polynomial dependence on temperature and pressure, the PR-EoS performs poorly in predicting those compositions even with a separately optimized BIC for the gas phase.
- As a potentially useful tool for the hydrogen energy community, we also derive purely parametric relations to match the experimental VLE data. Similar to the optimized PR EoS, such a parameterization can predict the aqueous compositions (e.g. hydrogen solubility) with a high accuracy but not the compositions of the $H_{2}$ – $H_{2}O$ gas phase in VLE.
- As an illustration of why VLE modeling is important in the context of hydrogen energy storage or exploration, we present hydrogen solubilities versus subsurface depth under conditions of hydrostatic equilibrium and a range of geothermal gradients. We highlight that the temperature dependence, in particular, is highly non-linear and non-monotonic. Hydrogen solubilities decrease up to temperatures of around 340 K and then increase. Depending on the geothermal gradient for a given formation, this happens at different depths and needs some care in estimating correctly. The relations presented in this work should help researchers and engineers with such estimates.
- Both hydrogen storage and natural hydrogen production scenarios typically also involve other common subsurface species such as $CO_{2}$ and $CH_{4}$. We provide predicted VLE compositions for $H_{2}O$-$CH_{4}$-$H_{2}$ and $H_{2}O$-$CO_{2}$-$H_{2}$ ternary mixtures, which exhibit more non-linear and non-monotonic pressure dependencies than the binary $H_{2}O$-$H_{2}$ system. This is due to the cross-association between $H_{2}O$ and polar-induced moments in $CH_{4}$ and $CO_{2}$. This behavior was observed experimentally for $H_{2}O$-$CH_{4}$ and $H_{2}O$-$CO_{2}$ mixtures and is accurately modeled by the CPA EoS [19], but cannot be captured by simpler EoS or correlations like Henry’s law.

In summary, the CPA equation of state, with specific terms to also capture cross-association with common molecules like $CO_{2}$, $CH_{4}$, and $H_{2}S$, provides a physically robust and accurate modeling tool to predict the phase behavior of hydrogen in societally important problems related to the storage and production of hydrogen from subsurface formations.

## Supporting information

S1 TableExperimental literature data [31–36] for hydrogen solubilities in water ($H_{2_{w,exp}},‰$) and water fractions in the gas phase (${H_{2}O}_{g,exp},‰$), which together fully specify the compositions of two-phase two-component VLE mixtures of water and hydrogen.(PDF)
