# Supplementary material for: Vapor-liquid equilibrium of water-hydrogen mixtures: A review of experimental data and modeling with a Cubic-Plus-Association Equation-of-State
Source: PLoS One. 2025 Dec 29;20(12):e0332157. doi: 10.1371/journal.pone.0332157 (PMC12747400; doi:10.1371/journal.pone.0332157)
Supplement: S1 Table — Experimental literature data [31–36] for hydrogen solubilities in water (H2w,exp,‰) and water fractions in the gas phase (H2Og,exp,‰), which together fully specify the compositions of two-phase two-component VLE mixtures of water and hydrogen. (PDF) [file pone.0332157.s001.pdf]

Vapor-Liquid Equilibrium of Water-Hydrogen Mixtures: A Review of Experimental Data and Modeling with a Cubic-Plus-Association Equation-of-State

Supplemental Information

Joachim Moortgat[1¶*]

[1] School of Earth Sciences, The Ohio State University, Columbus, Ohio, USA

* Corresponding author
Email: moortgat.1@osu.edu
¶ These authors contributed equally to this work.

Appendix

VLE Experimental Data

Table S1. Experimental literature data [1–6] for hydrogen solubilities in water ($H_{2w,\mathrm{exp}}$) and water fractions in the gas phase ($H_2O_{g,\mathrm{exp}}$), which together fully specify the compositions of two-phase two-component VLE mixtures of water and hydrogen.

T(K)	P(bar)	$H_{2w,\mathrm{exp}}$	$H_2O_{g,\mathrm{exp}}$
273.15	25.00	0.43	-
273.15	51.00	0.86	-
273.15	100.57	1.71	-
273.15	202.95	3.35	-
273.15	405.05	6.4	-
298.15	25.00	0.35	-
298.15	51.00	0.69	-
298.15	100.57	1.39	-
298.15	202.95	2.71	-
298.15	405.05	5.24	-
310.94	3.40	0.05	1.96
310.94	13.80	0.18	0.49
310.94	31.00	0.41	0.22
310.94	65.50	0.86	0.12
310.94	103.40	1.33	0.08
310.94	137.90	1.76	0.06
323.15	25.00	0.33	-
323.15	31.80	0.4	-
323.15	50.00	-	0.27
323.15	51.00	0.65	-
323.15	60.15	0.76	-
323.15	100.57	1.29	0.14
323.15	119.30	1.51	-
323.15	150.00	-	0.11

Continued on next page

Table S1. continued from previous page

T(K)	P(bar)	$H_{2w,\mathrm{exp}}$	$H_2O_{g,\mathrm{exp}}$
323.15	200.00	-	0.09
323.15	202.95	2.54	0.08
323.15	250.00	-	0.08
323.15	300.00	-	0.07
323.15	405.05	4.92	0.05
343.15	50.00	-	0.66
343.15	100.57	-	0.36
343.15	150.00	-	0.26
343.15	200.00	-	0.21
343.15	250.00	-	0.18
343.15	300.00	-	0.16
348.15	25.00	0.33	-
348.15	51.00	0.66	-
348.15	100.57	1.32	-
348.15	202.95	2.6	-
348.15	405.05	5.04	-
366.48	3.40	0.04	24.7
366.48	13.80	0.19	5.7
366.48	27.60	0.37	3.08
366.48	31.00	0.43	2.64
366.48	55.13	0.75	1.63
366.48	65.50	0.89	1.32
366.48	110.30	1.5	0.65
366.48	137.90	1.84	0.67
373.15	21.00	0.3	-
373.15	25.00	0.37	-
373.15	31.00	0.45	-
373.15	42.00	0.56	-
373.15	51.00	0.73	-
373.15	57.00	0.82	-
373.15	62.00	0.82	-
373.15	82.00	1.08	-
373.15	100.57	1.45	-
373.15	102.00	1.32	-
373.15	120.90	1.75	-
373.15	153.70	2.23	-
373.15	202.95	2.85	-
373.15	405.05	5.46	-
398.15	23.00	0.34	-
398.15	33.00	0.5	-
398.15	43.00	0.65	-
398.15	51.96	0.72	-
398.15	63.00	0.93	-
398.15	82.00	1.13	-
398.15	83.00	1.21	-
398.15	87.05	1.21	-
398.15	102.00	1.43	-
422.89	21.00	0.33	-
422.89	30.00	0.5	-

Continued on next page

Table S1. continued from previous page

T(K)	P(bar)	$H_{2w,\text{exp}}$	$H_2O_{g,\text{exp}}$
422.89	31.00	0.47	15.7
422.89	40.00	0.66	-
422.89	42.00	0.6	-
422.89	50.00	-	10.6
422.89	51.96	0.84	-
422.89	54.30	0.98	-
422.89	55.13	0.88	-
422.89	62.00	0.94	-
422.89	65.50	1.16	7.53
422.89	75.97	1.25	-
422.89	82.00	1.34	-
422.89	87.05	1.6	-
422.89	100.57	-	6.0
422.89	103.40	1.88	5.13
422.89	150.00	-	4.4
422.89	200.00	-	3.6
422.89	250.00	-	3.12
422.89	300.00	-	2.83
448.15	22.00	0.33	-
448.15	29.00	0.48	-
448.15	39.00	0.66	-
448.15	50.00	-	20.6
448.15	51.96	0.75	-
448.15	60.15	0.96	-
448.15	62.00	0.94	-
448.15	80.00	1.24	-
448.15	100.57	-	12.1
448.15	150.00	-	9.27
448.15	200.00	-	7.8
448.15	250.00	-	6.88
448.15	300.00	-	6.23
473.15	36.00	0.57	-
473.15	42.00	0.55	-
473.15	46.00	0.84	-
473.15	50.00	-	26.6
473.15	51.96	0.75	-
473.15	75.97	1.22	-
473.15	82.00	1.24	-
473.15	100.57	-	14.8
473.15	102.00	1.4	-
473.15	118.00	1.62	-
473.15	150.00	-	10.8
473.15	200.00	-	8.8
473.15	250.00	-	7.6
473.15	300.00	-	6.9

References

1. Chabab S, Theveneau P, Coquelet C, Corvisier J, Paricaud P. Measurements and predictive models of high-pressure H2 solubility in brine (H2O+ NaCl) for underground hydrogen storage application. International Journal of Hydrogen Energy. 2020;45(56):32206–32220.

2. Alanazi A, Bawazeer S, Ali M, Keshavarz A, Hoteit H. Thermodynamic modeling of hydrogen–water systems with gas impurity at various conditions using cubic and PC-SAFT equations of state. Energy Conversion and Management: X. 2022;15:100257.

3. Wiebe R, Gaddy V. The Solubility of Hydrogen in Water at 0, 50, 75 and 100 from 25 to 1000 Atmospheres. Journal of the American Chemical Society. 1934;56(1):76–79.

4. Pray HA, Schweickert C, Minnich BH. Solubility of hydrogen, oxygen, nitrogen, and helium in water at elevated temperatures. Industrial & Engineering Chemistry. 1952;44(5):1146–1151.

5. Rahbari A, Brenkman J, Hens R, Ramdin M, Van Den Broeke LJ, Schoon R, et al. Solubility of water in hydrogen at high pressures: a molecular simulation study. Journal of Chemical & Engineering Data. 2019;64(9):4103–4115.

6. Young C. IUPAC Solubility Data Series: Hydrogen and Deuterium; 1981; Vol. 5/6. vol. 5. Oxford, England: Pergamon Press; 1981.
